# Construction of a femininity score in the UK Biobank and its association with angina diagnosis prior to myocardial infarction

**DOI:** 10.1038/s41598-022-05713-x

**Published:** 2022-02-02

**Authors:** Anna Levinsson, Simon de Denus, Johanna Sandoval, Louis-Philippe Lemieux Perreault, Joëlle Rouleau, Jean-Claude Tardif, Julie Hussin, Marie-Pierre Dubé

**Affiliations:** 1 Beaulieu-Saucier Université de Montréal Pharmacogenomics Centre, Montreal, QC Canada; 2 Montreal Heart Institute, Montreal, QC Canada; 3 Faculty of Medicine, Université de Montréal, Montreal, QC Canada; 4 Division of Social and Cultural Psychiatry, McGill University, Montreal, QC Canada; 5 Faculty of Pharmacy, Université de Montréal, Montreal, QC Canada; 6 Faculty of Arts and Sciences, Université de Montréal, Montreal, QC Canada

**Keywords:** Cardiology, Health care, Medical research

## Abstract

Gender captures social components beyond biological sex and can add valuable insight to health studies in populations. However, assessment of gender typically relies on questionnaires which may not be available. The aim of this study is to construct a gender metric using available variables in the UK Biobank and to apply it to the study of angina diagnosis. Proxy variables for femininity characteristics were identified in the UK Biobank and regressed on sex to construct a composite femininity score (FS) validated using tenfold cross-validation. The FS was assessed as a predictor of angina diagnosis before incident myocardial infarction (MI) events. The FS was derived for 315,937 UK Biobank participants. In 3059 individuals with no history of MI at study entry who had an incident MI event, the FS was a significant predictor of angina diagnosis prior to MI (OR 1.24, 95% CI 1.10–1.39, P < 0.001) with a significant sex-by-FS interaction effect (P = 0.003). The FS was positively associated with angina diagnosis prior to MI in men (OR 1.37, 95% CI 1.19–1.57, P < 0.001), but not in women. We have provided a new tool to conduct gender-sensitive analyses in observational studies, and applied it to study of angina diagnosis prior to MI.

## Introduction

Scientific research conventions dictate that epidemiological and clinical studies will include data analyses that are sensitive to differences in sex by either adjusting for or by conducting analyses stratified according to sex. However, biological sex does not fully explain the sex discrepancies in cardiovascular care^1–3^. There is an additional social component to sex that can be captured through gender, defined here as the social construct counterpart to sex^4^. Gender can be further broken down into four components: the gender role, which relates to social expectations based on appearance and behavior; the gender identity, which is the gender that the individual identifies with; gender perception, which is how an individual’s gender is perceived by others in their social roles; and finally institutional gender, which is how an individual is responded to by social institutions based on gender presentation^4–6^. In order to reach a better understanding of the relative contribution of both sex and gender components to research phenotypes, measures of both sex and gender need to be considered in analyses.

It is useful to clearly state definitions and distinctions of possibly ambiguous terms. Therefore, in this article: *man* and *woman* refers to biological sex characteristics, e.g. sex chromosomes and hormonal levels; *masculine* and *feminine* refers to the individual’s gender identification, an agglomeration of sex, masculinity and femininity characteristics, as well as are collective terms representing the stereotypical characteristics of respective gender role. In describing the characteristics of femininity and masculinity, we are not describing ‘women’ or ‘men’, but rather attempting to capture stereotypical gender role constructions that are subject to geographical, temporal and conceptual fluctuations according to social norms.

While cardiovascular research has been at the forefront of studies on sex differences, no standard quantification of gender has yet been developed^7^. One major roadblock is that there is no consensus in medical science on how to quantify gender, with little research published on the subject. The Bem sex role inventory (BSRI) was published in 1974 and is still the most commonly used tool to measure gender^8^. The BSRI is a psychological instrument that asks the patient to rate, on a Likert scale from one to seven, how much they identify with up to 60 numbered terms that are connected with stereotypical masculine and feminine social gender roles, e.g. ‘athletic’, ‘aggressive’ and ‘willing to take a stand’ for masculinity, and ‘yielding’, ‘tender’, and ‘loves children’ for femininity. Hunt et al. showed in a small Scottish sample (n = 1417) of individuals born in the 1930’s and aged on average 55 years at time of interview, that femininity as measured by the BSRI was associated with lower coronary heart disease death in men but not in women^9^. While the BSRI has shown good validity, one of its drawbacks is that it relies on psychological research methods using self-reported, self-identification, and self-assessment only. Such emphasis on self-perception understates the complementary aspects of gender role and gender presentation^10^. Applied to medicine and health care, gender role and gender presentation are as important as the self-identified gender identity, since gender as perceived by others may influence decisions made by a treating physician, medication prescriptions, and even which diseases are considered for differential diagnosis. For example, while men presenting with angina or myocardial infarction (MI) are likely to show classic symptoms such as pain in the left arm, women are more likely to present with ‘atypical’ symptoms such as jaw or stomach pain^11,12^. The deviation from the expected ‘norm’ may undermine women's chances of obtaining equal care. Furthermore, there are also important gender differences in the use of, and approach to, health care services^13–15^. Another drawback to using the BSRI is the age of the instrument given the temporality of gender roles. To investigate current gender differences in coronary heart disease (CHD) diagnoses, an updated and quantifiable measure of femininity and masculinity is called for^13–15^.

A composite measure for gender in a cardiovascular disease cohort was presented in 2015^16^. This measure included a number of psychosocial and demographic variables in addition to the BSRI. Unfortunately, most health-related cohort studies, of cardiovascular or other diseases, do not include the BSRI questionnaire. It is therefore of interest to develop an alternative gender metric which does not rely on the BSRI but rather on variables available from observational cohort studies of a broad spectrum of phenotypes. Suitable variables for the construction of a femininity measure can be found in self-reported questionnaires on lifestyle, psychosocial questionnaires, as well as from observational and demographic data.

In the present study, we aimed to construct a culturally appropriate yet objective quantification of femininity using variables available without use of the BSRI. The objective of our constructed femininity score (FS) is to enable and promote the conduct of gender-aware epidemiological studies to explain variations in diagnoses and disease outcomes, including CHD, among men and women that are not fully accounted for by sex alone. As a first application of the FS, we tested it as a predictor of angina diagnosis before a myocardial infarction event, under the hypothesis that stereotypical femininity as measured with the FS may not show the same phenotype associations in men and women.

## Results

Descriptive data for the UK Biobank including the selected FS candidate variables (work status, education, risk-taking, self-reported depression, neuroticism and the control variable birth year) are presented in Table 1. Data is partially missing for all femininity score candidate variables except for sex and birth year, with the most limiting factor being the neuroticism score. Participants who had all FS component variables were born in 1934–1971. Fifty-three percent of participants in the UK biobank were women. Risk-taking behaviour was reported more by men (35%) compared to women (20%). The most marked difference between the sexes was for employment status ‘taking care of home and/or family’ which was reported by four of every hundred women but only five per thousand men. The majority of men and women reported being gainfully employed, and around a third reported retirement. More men than women had incident diagnoses of MI (1.7% vs. 0.5% respectively).

**Table 1:** Descriptive statistics for individuals with calculated femininity score.

	Overall	Women	Men	p-value
	N = 315,937	n = 166,706 (53%)	n = 149,231 (47%)	
Age, years [mean (SD)]	56.6 (8.0)	56.4 (7.9)	56.9 (8.0)	< 0.001
Height, cm [mean (SD)]	169.1 (9.2)	162.9 (6.2)	176.1 (6.7)	< 0.001
Waist-hip ratio [mean (SD)]	0.87 (0.09)	0.82 (0.07)	0.93 (0.06)	< 0.001
BMI, kg/m^2^ [mean (SD)]	27.4 (4.7)	27.0 (5.1)	27.8 (4.2)	< 0.001
SBP, mmHg [mean (SD)]	137.9 (18.5)	135.1 (19.0)	141.1 (17.3)	< 0.001
DBP, mmHg [mean (SD)]	82.3 (10.1)	80.6 (9.9)	84.1 (10.0)	< 0.001
Prevalent hypertension (%)	292,512 (92.6)	156,326 (93.8)	136,186 (91.3)	< 0.001
Townsend deprivation index [mean (SD)]	− 1.56 (2.94)	− 1.58 (2.89)	− 1.54 (2.98)	0.002
Current smoking (%)	31,228 (9.9)	14,284 (8.6)	16,944 (11.4)	< 0.001
**Biomarker measurements**				
Triglycerides, mmol/L [mean (SD)]	1.75 (1.03)	1.55 (0.86)	1.98 (1.15)	< 0.001
Total cholesterol, mmol/L [mean (SD)]	5.71 (1.14)	5.89 (1.12)	5.51 (1.12)	< 0.001
HDL, mmol/L [mean (SD)]	1.45 (0.38)	1.60 (0.38)	1.29 (0.31)	< 0.001
LDL, mmol/L [mean (SD)]	3.57 (0.87)	3.64 (0.87)	3.49 (0.86)	< 0.001
HbA1c, mmol/mol [mean (SD)]	35.9 (6.4)	35.6 (5.7)	36.2 (7.1)	< 0.001
**CV outcomes from hospital records**				
MI—incident (%)	3292 (1.0)	814 (0.5)	2478 (1.7)	< 0.001
Any angina—prevalent (%)	7289 (2.3)	2342 (1.4)	4947 (3.3)	< 0.001
Any angina—incident (%)	9105 (2.9)	3067 (1.8)	6038 (4.0)	< 0.001
Angina before incident MI (% of MI cases)	564 (17.1)	129 (15.8)	435 (17.6)	0.286
**Femininity score component variables**				
Education (%)				
NVQ/HND/HNC/equiv	40,334 (12.8)	15,848 (9.5)	24,486 (16.4)	< 0.001
College or University deg	106,733 (33.8)	54,204 (32.5)	52,529 (35.2)	
None of the above	49,942 (15.8)	26,257 (15.8)	23,685 (15.9)	
A levels/AS levels/equiv	37,127 (11.8)	20,985 (12.6)	16,142 (10.8)	
CSE/equiv	11,103 (3.5)	6321 (3.8)	4782 (3.2)	
Other	28,840 (9.1)	16,705 (10.0)	12,135 (8.1)	
O levels/GCSEs/equiv	41,858 (13.2)	26,386 (15.8)	15,472 (10.4)	
Employment (%)				
Unemployed	4155 (1.3)	1300 (0.8)	2855 (1.9)	< 0.001
Unable to work due to sickness/disability	9122 (2.9)	3892 (2.3)	5230 (3.5)	
Paid employment/self-employed	186,282 (59.0)	94,075 (56.4)	92,207 (61.8)	
None of the above	106,530 (33.7)	58,844 (35.3)	47,686 (32.0)	
Full- or part-time student	624 (0.2)	431 (0.3)	193 (0.1)	
Unpaid or volunteer work	1344 (0.4)	968 (0.6)	376 (0.3)	
Taking care of home and family	7880 (2.5)	7196 (4.3)	684 (0.5)	
Depression (%)				
Not at all	246,012 (77.9)	125,010 (75.0)	121,002 (81.1)	< 0.001
Several days	56,602 (17.9)	33,795 (20.3)	22,807 (15.3)	
More than half the days	7964 (2.5)	4727 (2.8)	3237 (2.2)	
Nearly every day	5359 (1.7)	3174 (1.9)	2185 (1.5)	
Risk-taking (%)				
Yes	85,416 (27.0)	33,244 (19.9)	52,172 (35.0)	< 0.001
No	230,521 (73.0)	133,462 (80.1)	97,059 (65.0)	
Neuroticism score [mean (SD)]	4.06 (3.24)	4.52 (3.23)	3.55 (3.18)	< 0.001
Birth year, calendar year [mean (SD)]	1951.45 (7.96)	1951.69 (7.88)	1951.17 (8.04)	< 0.001
Vitamin supplement use (%)				
No	216,287 (68.5)	107,165 (64.3)	109,122 (73.1)	< 0.001
Yes	98,963 (31.3)	59,307 (35.6)	39,656 (26.6)	
Missing	685 (0.2)	233 (0.1)	452 (0.3)	
FS standardized [mean (SD)]	0.00 (1.00)	0.26 (1.02)	− 0.29 (0.89)	< 0.001

Unless stated otherwise, variables were measured at baseline. *A-level* Advanced Level, *AS-Level* Advanced Subsidiary, *BMI* Body Mass Index, *CSE* Certificate of Secondary Education, *DBP* diastolic blood pressure, *GCSE* General Certificate of Secondary Education, *HbA1c* Hemoglobin A1c, *HDL* high-density lipoprotein, *HNC* Higher National Certificate, *HND* Higher National Diploma, *LDL* low-density lipoprotein, *NVQ* National Vocational Qualification, *SBP* systolic blood pressure.

### Femininity score in the UK Biobank

**Figure 1:**
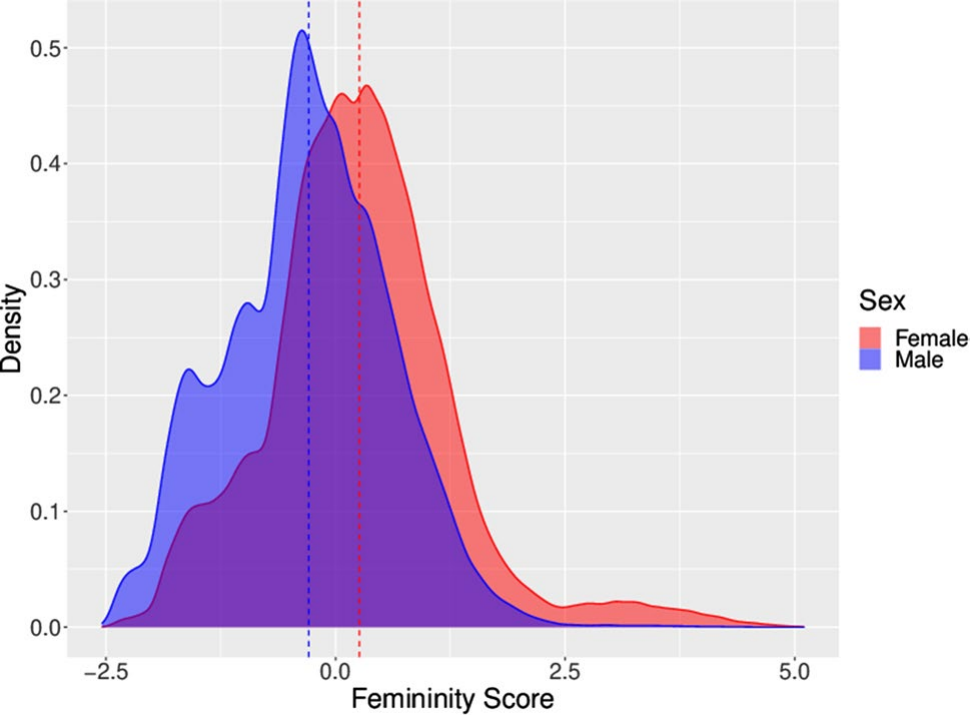
Distribution of standardized femininity score (FS) in women and men.

We selected five candidate variables based on their identification in the literature as femininity traits. All five variables were significantly associated with sex in univariate models and remained significant predictors of sex with p-values < 0.001 in a model including all five variables as well as birth year (Table 2). A tenfold cross validation of the model gave an RMSE = 0.47 ~ 0.50, indicating good model performance. The estimated β-coefficients calculated from the full population were used as coefficients for the FS algorithm to calculate a FS for each individual (last column of estimates, Table 2). The FS was standardized in the full population, and the distribution of the FS in women and men is shown in Fig. 1. The final FS formula was: FS= - 5.08470187338465 + 0.19807196894789*education + 0.042026387488911*work status - 0.006756305442782*depression + 0.173153140282766*risk taking + 0.020797148428804*neuroticism + 0.002134711107064*birthyear

**Table 2:** Parameter estimates from univariate and multivariable linear regressions on outcome sex (women vs men).

Dependent variable	Sex		Sex		Sex		Sex		Sex		Sex		Sex	
Predictors	Estimate (SE)	p-value	Estimate (SE)	p-value	Estimate (SE)	p-value	Estimate (SE)	p-value	Estimate (SE)	p-value	Estimate (SE)	p-value	Estimate (SE)	p-value
(Intercept)	0.69	< 0.001	0.53	< 0.001	0.54	< 0.001	0.61	< 0.001	0.57	< 0.001	4.36	< 0.001	− 5.09	< 0.001
Educational level	0.22 (0.003)	< 0.001											0.2	< 0.001
													(0.0043)	
Work status			0.05 (0.001)	< 0.001									0.04	< 0.001
													(0.0007)	
Depression					0.05 (0.001)	< 0.001							− 0.01	< 0.001
													(0.0017)	
Risk-taking							0.19 (0.002)	< 0.001					0.17	< 0.001
													(0.0019)	
Neuroticism									0.02 (0.000)	< 0.001			0.02	< 0.001
													(0.0003)	
Birth year											0 (0.000)	< 0.001	0.00	< 0.001
													(0.0001)	
Observations (N)	314,551		314,551		314,551		314,551		314,551		314,551		314,551	

*SE* standard error.

### FS as a predictor of angina diagnosis before an MI event

Analyses of angina diagnosis before an MI event were limited to 3059 individuals (2274 men and 785 women) with no history of MI at baseline, and who experienced at least one MI during the follow-up period of median 4.95 years. Overall, the FS was significantly associated with angina diagnosis before MI (OR 1.24, 95% CI 1.10–1.39, P < 0.001). We saw a significant sex*FS interaction effect (OR 1.53, 95% CI 1.16–2.02, P = 0.003) (Table 3). When stratified by sex, the FS was positively associated with angina diagnosis before an MI event in men (OR 1.37, 95% CI 1.19–1.57, P < 0.001), but the effect was not significant in women (OR = 0.90, 95% CI 0.70–1.15, P = 0.41).

**Table 3:** Logistic regression for angina diagnosis prior to the occurrence of myocardial infarction (MI) in individuals with incident MI and no history of MI at baseline.

Variable	Angina before incident MI			Angina before incident MI			WOMEN: Angina before incident MI			MEN: Angina before incident MI		
	Odds ratios	95% CI	p-value	Odds ratios	95% CI	p-value	Odds ratios	95% CI	p-value	Odds ratios	95% CI	p-value
Femininity score	1.24	1.10–1.39	< 0.001	0.90	0.70–1.15	0.39	0.90	0.70–1.15	0.41	1.37	1.19–1.57	< 0.001
Age	1.07	1.05–1.09	< 0.001	1.07	1.05–1.09	< 0.001	1.08	1.04–1.12	< 0.001	1.07	1.05–1.09	< 0.001
Sex	1.15	0.90–1.47	0.27	1.12	0.88–1.43	0.36						
Sex*FS				1.53	1.16–2.02	0.003						
Observations (N)	3059			3059			785			2274		

*CI* confidence interval, *MI* myocardial infarction.

### Sensitivity analyses

To assess whether the positive association between FS and angina diagnosis prior to MI observed in men could be explained by healthier lifestyle in individuals with higher FS, we tested the association of FS with smoking and with use of vitamin supplements. The FS was negatively associated with current smoking in the full sample (OR 0.96, 95% CI 0.95–0.97, P < 0.001), as well as in women and men in sex-stratified analyses. Vitamin supplement use was negatively associated with FS in women only, OR 0.98, 95% CI 0.97–0.99, P = 0.001. (Supplementary Tables 2–3) The robustness of the association between FS and angina diagnosis before MI was tested in high and low age groups and high and low Townsend deprivation index groups, showing consistency in the estimated associations across age- and deprivation level groups. (Supplementary Tables 4–5).

## Discussion

In the present study, we have constructed a femininity score (FS) from selected variables available from the UK Biobank, a large epidemiological cohort study, in order to derive a quantitative measure of stereotypical femininity as a social construct to inform medical research. The FS was derived from the regression of sex on five variables considered indicative of the stereotypical feminine gender role and gender identity, selected from the UK Biobank questionnaire variables: work status, level of education, neuroticism, self-reported depressed mood, and self-reported risk-taking behaviour, with a model correction for birth year. The variables were selected based on literature of perceived gender characteristics. The derived FS opposes stereotypical masculinity and femininity traits and provides higher scores for feminine traits. We saw a different effect of the FS in men and women for the prediction of angina diagnosis prior to an MI event, with a higher FS being predictive of angina diagnosed before MI in men but not in women.

The choices of variables used to characterize gender were informed by the literature. Risk-taking was previously reported to be one of eleven factors that characterize stereotypical masculinity and was used reverse-coded (i.e. femininity associated with less risk-taking)^17,18^. The employment status variable was used as a proxy for care of the home and family as stereotypically attributed to women. The different skills valued for the stereotypical nurturing mother compared to the “breadwinner” father has contributed to the attribution of higher levels of education as a stereotypical masculine trait (i.e. stereotypical femininity has been associated with less education)^19,20^. Neuroticism is a personality trait in the Big Five personality theory, for which women tend to score higher^18^. People who score high on neuroticism are considered more likely to act out emotion, a stereotypical feminine characteristic^21,22^.

The FS has enabled us to study the effect of stereotypical femininity on the diagnosis of angina prior to an MI event in both men and women, and we found a significantly different effect according to sex, supporting our original hypothesis that stereotypical femininity may not show the same phenotype associations in men and women. The results indicate that patients’ femininity traits may contribute to the diagnosis of angina differently in the two sexes. Angina is a strong risk factor for MI. From primary prevention to first cardiovascular event, and from secondary prevention to death, it has been shown that women are more likely to be underdiagnosed when presenting with angina or myocardial infarction^11,12,23–27^. It has been suggested that this discrepancy is at least partly due to gender role stereotypes^28^. We had expected to see a negative association between the FS and angina diagnosis in women, which was not found. However, the finding of a positive association between the FS and angina diagnosis in men is compelling. Femininity has been associated with health-seeking behavior, i.e. contacting health services with less delay than more stereotypically masculine patients^29^. It may be speculated that feminine traits may involve a healthier lifestyle or greater health-seeking behavior characteristics leading to higher chances of angina diagnosis. This is partly supported by the sensitivity analyses using variables to capture unhealthy lifestyle and health-seeking behavior (*smoking* and *use of vitamin supplement* respectively) which showed a negative association between the FS and ever-smoking in the overall sample, in men and in women. This result was expected, given that women’s tobacco consumption has always been lower than men’s consumption^30^. Nonetheless, the result is opposing that reported by Hunt et al.^30^, who reported a weak association between femininity as measured by the BSRI and smoking in men and a positive association in women. However, the Hunt et al. study was conducted in west Scotland while the present FS was calculated for a mostly English population. Differences in femininity may exist between the two populations. However, a higher FS was associated with less vitamin supplement use in women only, results were non-significant for men and the overall population. While the prevalence of woman consumers of vitamin supplements is higher relative to men, the negative association between femininity and vitamin use may be due to temporal trends. A study of US adults showed a steady decrease in woman vitamin supplement use between 2005 and 2010^31^, i.e. the time period when UK biobank participants filled out baseline questionnaires.

A notable strength of the developed model is that because the data were collected as part of a cohort study not specifically designed for the development of the FS, the score components are not biased by preconceptions about a metric of gender, although susceptibility to reporting bias remains. The proposed FS is intended as a proof of concept and we expect that the FS will benefit from future revisions and adaptations to different cohorts and different social norms. Recently, Lacasse et al.^32^ published the GENDER Index (GI) based on variables from the Canadian Community Health Survey (CCHS) for participants living in the Canadian province Quebec. There are some noteworthy differences between the GI and the FS. In particular, the dataset used to develop the FS has more than 10 times the number of participants than that of the GI, and while the GI relies mainly on socio-economic and demographic variables, allowing for a measure of institutionalized gender, the FS enables a measure of the gender roles. There also are some noteworthy advantages of the FS over the traditional BSRI in the context of health research, which is attributable to the value of a gender metric to capture a person’s exposure to health risk factors. In epidemiological research, gender can be considered a social determinant of health^33^. Examples of social determinants of health are values, attitude, knowledge and neighbourhood conditions, which are captured by the components of the FS. Because the BSRI captures femininity traits through self-evaluation, it provides a less direct measure which is more susceptible to additional sources of bias. The major strength of the present study is in the demonstration that the use of a few selected variables enables the derivation of a FS, an approach that can be applied to a wider range of cohorts than measures based on the BSRI. Nonetheless, Hunt et al.^9^ showed that femininity measured by the BSRI was negatively associated with CHD death in men, which is in line with the results shown here, including the smoking sensitivity analyses, suggesting that the FS is indeed measuring a similar conception of femininity.

It is of worth to note that the UK Biobank is 54% women and enables the construction of a gender score applicable to both sexes. To promote gender equity in health, both the question of the woman to man participation rate, and that of the quality of participation of individuals with different gender identities must be addressed. A gender-sensitive approach is thus called for, given that health outcomes are associated with gender. By using information typically collected in cohort study designs, as presented here, the opportunities to study gender are maximised.

### Limitations

The study has some limitations worth mentioning. In particular, the selected variables reflect the gender roles of the participants’ generation, consequently, gender scores will need to be recalibrated as patients’ birth year changes. While some variables of behavior, such as reported depression, are converging in men and women in Western societies, other variables may diverge according to sex over time. For this reason, birth year is a more precise variable than age, for which the year of data collection must be included in order to contextualise and interpret the meaning of age. Nevertheless, all regressions in this study were adjusted for age to correct for confounding. A limitation of the FS is that while it is developed using a sample from the general British population, which is representative of a Western high-income nation, it is not sensitive to cultural differences that can exist in such communities, such as those that may impact the construct of stereotypical femininity between nations such as France and the UK. In addition, the birth year range of participants with complete data for the FS variables is limited to 1934–1971, who were aged 39–73 years at baseline, and results may not be valid for younger or older population groups. However, upper middle age is the most common age for onset of cardiovascular disease and where the incidence of chronic disease increases. Thus, the age span covered by the UK Biobank is very suitable for testing the FS as a predictor of CHD. Further, the sensitivity analyses by age group showed consistency in the association between FS and angina diagnosis before MI across age groups. Another limitation includes our reliance on angina obtained from ICD codes from hospitalization events. As not everyone presenting and diagnosed with angina will need hospitalisation, this may introduce information bias. While the addition of self-reports of angina at baseline would enable to capture both in- and out-patient care, self-reporting is subject to recollection bias, and is sensitive to the time difference between baseline and the MI event date. We have verified that the FS was not significantly associated with the total number of hospitalisations for any cause. An additional limitation is that the score has not yet been validated in another population, and the results are not ready to inform changes in clinical practice. Finally, alternative methods to capture femininity and masculinity using non-linear models may provide better precision, which could be explored in future work.

On a different note, the application of the FS presented here does not capture a non-normative alignment of gender and sex in cis-individuals directly. Indeed, it may be of interest to assess the health consequences of a misalignment in gender and sex on the gender perception and queerness, however a different metric or at least a different use of the FS metric would be needed to this end.

## Conclusions

In conclusion, we presented a FS derived from a weighted measure of socio-cultural and economic risk factors and applied it to the study of cardiovascular outcomes in an epidemiological cohort study. We found that the FS is a predictor of angina diagnosis prior to MI event, providing information additional to what is captured by sex alone. Our results suggest that higher stereotypical femininity scores are associated with higher probability of diagnosis with angina prior to an MI event in men but not in women.

The continued pursuit of studies of gender differences and effects is warranted to inform future research directions and health policy.

## Methods

### UK Biobank

The UK Biobank has been described in detail in previous publications, e.g.^34,35^ as well as on the UK Biobank website (https://www.ukbiobank.ac.uk/). In summary, 502,619 participants were recruited from the surrounding area of 22 participating health centres in England, Scotland and Wales between 2006 and 2010. At time of recruitment, participants gave broad written consent to the use of their pseudo-anonymized data including biological samples, questionnaires and medical records for health research.

### Study variables

To build the femininity score, we first identified stereotypically feminine traits from the literature with matching information collected in the UK Biobank. The targeted information included family values, caring traits, and neuroticism, which have a positive correlation with stereotypical femininity, and high education achievement, career advancement, and risk-taking which have a negative correlation^2,36–40^. In addition, self-reporting of depression is associated with femininity and is less common in men^41^, and femininity has been associated with greater self-disclosure^42^. Information on femininity variables was derived from the UK Biobank initial assessment visit (2006–2010) touchscreen questions (refer to Supplementary Table 1 for additional information).

We defined biological sex, the dependent variable for the regression estimating the variable weights, based on sex chromosomes. Demographic and lifestyle information was taken from baseline questionnaires (first assessment visit, i.e. Instance 0). All variables except for ‘Current employment status’, ‘Education’ and ‘Risk-taking’ used the coding made by the UK Biobank in the direction of femininity, i.e. all variable coding is based on the assumption that women are on average more feminine than men. (Supplementary Table 1) As an example, coding for Risk-taking (Data-field 2040) was reversed to 1 = “No”, 0 = “Yes” Self-reported depression was measured on a rating scale, known as Likert scale, as frequency of depressed mood in the last 2 weeks (Data-field 2050) with answers coded as “Not at all” = 1, “Several days” = 2, “More than half of the days” = 3 and “Nearly every day” = 4. Education (Data-field 6138) was captured in 6 categories, which we coded according to the proportion of women to men in each category: “NVQ or HND or HNC or equivalent” = 0.51, “College or University degree” = 0.84, “CSEs or equivalent” = 1.17, “A levels/AS levels or equivalent” = 1.04, “O levels/GCSEs or equivalent” = 1.50, “Other professional qualifications e.g.: nursing, teaching = 1.29, and “None of the above” = 0.98. When a participant selected several educational levels as response, the FS was calculated using the coding for the highest educational level indicated. The 7 captured current employment status categories (Data-field 6142) were also scored according to the proportion of women to men in each category. We scored the categories: “Unemployed” = 0.45, “Unable to work because of sickness or disability” = 0.69, “In paid employment or self-employed” = 0.90, “Full or part-time student” = 1.66, “Doing unpaid or voluntary work” = 2.19, “Retired” = 1.13, “Full or part-time student” = 1.66, and “Looking after home and/or family” = 9.63. For individuals who reported more than one employment status, the FS was calculated using the coding of the employment status with the highest femininity weight. Neuroticism (Data-field 20,127) is a derived summary score, based on 12 neurotic behaviour domains as reported from data-fields 1920, 1930, 1940, 1950, 1960, 1970, 1980, 1990, 2000, 2010, 2020 and 2030 as used in previously published analyses^43^. Neuroticism summarises the number of “Yes” answers across these twelve questions and was coded as a continuous variable with range 0–12. The variable Birth year (Data-field 34) was included to control for the evolution of gender role stereotypes over time.

The variable MI was defined from ICD10 code I21 or ICD9 codes: 410, 412, 411.0, 429.79 in the hospitalization records or in the death records. Angina was defined based on the hospitalization records ICD10 code I20 or ICD9 code 413. For all outcomes, prevalent events were events that occurred before, and incident events after, a participant’s baseline date. The occurrence of an angina diagnosis prior to an incident MI was defined based on hospitalisation codes for angina that have occurred in a hospitalisation spell prior to that of the incident MI event.

The study was approved by the Montreal Heart Institute research ethics committee and complies with the Declaration of Helsinki.

### Statistical analyses

Under the assumption that women overall project more feminine characteristics than men, sex was used as the dependent variable for the regression used for modelling the FS. Selected FS component variables were coded in the direction of a positive association with femininity and were tested in univariate linear models for association with sex. The variables were first tested for significant univariate model association with sex (P < 0.05). All variables were thereafter jointly entered into a multiple linear regression model predicting sex. The performance of the resulting model was validated using tenfold cross validation. The estimated intercept and β-coefficients were used to calculate a femininity score for each participant according to the formula:

$$FS = intercept + \beta_{1} *education + \beta_{2} *work \, status + \beta_{3} *depression + \beta_{4} *risk {-}taking + \beta_{5} *neuroticism + \beta_{6} *birth \, year$$

Except where noted otherwise, analyses were performed using R version 3.6.0^44^ in Rstudio version 1.2.5042^45^.

### Femininity Score (FS) as a predictor of angina diagnosis

To assess whether the FS can contribute to the noted underdiagnosis of angina in women, above and beyond the effect of sex, we regressed ‘diagnosis of angina before incident MI event’ derived from hospitalization codes and baseline self-reporting on the base model with independent variables sex and FS, in 3,059 individuals with no history of MI at baseline, and who had an incident MI event, followed by sex-stratified analyses. Incident MI events were defined as occurring during the follow-up period of median 4.95 years in participants free of MI at baseline. Angina diagnoses originated from hospitalisation records, which included the date of the diagnosis; earliest recorded angina occurred on June 30, 2007, and the last on December 29, 2015. Baseline questionnaires were filled out between March 24, 2006 and September 29, 2010. Additional regression models were tested in the sample stratified by sex and interaction terms for FS*sex were introduced. Vitamin supplement use and smoking may be considered gendered variables^32,33^. In sensitivity analyses, vitamin supplement use and smoking status were regressed on age and FS in all individuals with FS data (n = 315,937) in the full sample and stratified by sex. Vitamin supplement use was expected to be positively associated with femininity and smoking negatively associated with femininity. The robustness of the association between FS and angina diagnosis before MI was tested in high and low age groups (cut-off median age in individuals with incident MI 62 years) and high and low Townsend deprivation index groups (cut-off mean deprivation level − 1.56).

All methods were carried out in accordance with relevant guidelines and regulations.

## Supplementary Information

Supplementary Tables.
