# Supplementary material for: Construction of a femininity score in the UK Biobank and its association with angina diagnosis prior to myocardial infarction
Source: Sci Rep. 2022 Feb 2;12:1780. doi: 10.1038/s41598-022-05713-x (PMC8810762; doi:10.1038/s41598-022-05713-x)
Supplement: Supplementary file 1 — Supplementary Tables. [file 41598_2022_5713_MOESM1_ESM.docx]

**Construction of a femininity score in the UK Biobank and its association with angina diagnosis prior to myocardial infarction**

**Anna Levinsson, Simon de Denus, Johanna Sandoval, Louis-Philippe Lemieux Perreault, Joëlle Rouleau, Jean-Claude Tardif, Julie Hussin, Marie-Pierre Dubé**

**SUPPLEMENTAL MATERIAL**

**Supplementary Table 1.** Coding of UK Biobank variables for the femininity score

| **UKB Data field** | **Variable** | **UKB coding** | **Our coding** | **Data source** |
| --- | --- | --- | --- | --- |
| 34 | Birth year | Year 1934-1971 | continuous 1934 - 1971 | Acquired from central registry, updated by participant |
| 2040 | Risk-taking | 0 = No | 0 = Yes | Initial assessment visit (2006-2010) touchscreen question "Would you describe yourself as someone who takes risks?"  If the participant activated the Help button they were shown the message: “Work through these questions quickly and do not think about the exact meaning of the question” |
|  |  | 1 = Yes | 1 = No |  |
| 2050 | Depression | 1 = “Not at all” | 1 = “Not at all” | Initial assessment visit (2006-2010) touchscreen question "Over the past two weeks, how often have you felt down, depressed or hopeless?"  If the participant activated the Help button they were shown the message: “Answer this question thinking about the past 2 weeks. If you are unsure, please provide an estimate or select Do not know.” |
|  |  | 2 = “Several days” | 2 = “Several days” |  |
|  |  | 3 = “More than half of the days” | 3 = “More than half of the days” |  |
|  |  | 4 = “Nearly every day” | 4 = “Nearly every day” |  |
| 6138 | Education | 1 = College or University degree | 0.51 = NVQ or HND or HNC or equivalent | Initial assessment visit (2006-2010) touchscreen question "Which of the following qualifications do you have? (You can select more than one)"  If the participant activated the Help button they were shown the message: “A levels/AS levels and equivalent includes the Higher School Certificate; O levels/GCSEs and equivalent includes the School Certificate.” |
|  |  | 2 = A levels/AS levels or equivalent *(Year 12-13, age 16-18)* | 0.84 = College or University degree |  |
|  |  | 3 = O levels/GCSEs or equivalent *(Year 11, age 15)* | 0.98 = None of the above |  |
|  |  | 4 = CSEs or equivalent *(similar to O-levels, only used 1965-87)* | 1.17 = CSEs or equivalent |  |
|  |  | 5 = NVQ or HND or HNC or equivalent *(vocational training)* | 1.04 = A levels/AS levels or equivalent |  |
|  |  | 6 = Other professional qualifications eg: nursing, teaching | 1.50 = O levels/GCSEs or equivalent |  |
|  |  | -7 = none of the above | 1.29 = Other professional qualifications eg: nursing, teaching |  |
| 6155 | Vitamin supplement use | 1 = Vitamin A | 0 = No | Initial assessment visit (2006-2010) touchscreen question "Do you regularly take any of the following? (You can select more than one answer)" |
|  |  | 2 = Vitamin B | 1 = Yes |  |
|  |  | 3 = Vitamin C |  |  |
|  |  | 4 = Vitamin D |  |  |
|  |  | 5 = Vitamin E |  |  |
|  |  | 6 = Folic acid or Folate (Vitamin B9) |  |  |
|  |  | 7 = Multivitamins =/- minerals |  |  |
|  |  | -7 = none of the above |  |  |
| 6142 | Current employment status | 1 = In paid employment or self-employed | 0.45 = Unemployed | Initial assessment visit (2006-2010) touchscreen question "Which of the following describes your current situation? (You can select more than one answer)"  The following checks were performed:  If the participant activated the Help button they were shown the message: “If more than one situation applies, select all that are appropriate.” |
|  |  | 2 = Retired | 0.69 = Unable to work because of sickness or disability |  |
|  |  | 3 = Looking after home and/or family | 0.90 = In paid employment or self-employed |  |
|  |  | 4 = Unable to work because of sickness or disability | 1.13 = Retired |  |
|  |  | 5 = Unemployed | 1.66 = Full or part-time student |  |
|  |  | 6 = Doing unpaid or voluntary work | 2.19 = Doing unpaid or voluntary work |  |
|  |  | 7 = Full or part-time student | 9.63 = Looking after home and/or family |  |
| 20116 | Smoking status | 0 = Never | 0 = Never or Previous | This field summarises the current/past smoking status of the participant derived from the Initial assessment visit (2006-2010) touchscreen questionnaire. |
|  |  | 1 = Previous | 1 = Current |  |
|  |  | 2 = Current |  |  |
| 20127 | Neuroticism score | * See below score 1-12 | + Score = + Femininity | This is an externally derived summary score of neuroticism, based on 12 neurotic behaviour domains as reported from fields 1920, 1930, 1940, 1950, 1960, 1970, 1980, 1990, 2000, 2010, 2020 and 2030 from the touchscreen questionnaire at the initial assessment visit (2006-2010). Participants were assessed for twelve domains of neurotic behaviours via the touchscreen questionnaire. Questions included:  Does your mood often go up and down?  Do you ever feel 'just miserable' for no reason?  Are you an irritable person?  Are your feelings easily hurt?  Do you often feel 'fed-up'?  Would you call yourself a nervous person?  Are you a worrier?  Would you call yourself tense or 'highly strung'?  Do you worry too long after an embarrassing experience?  Do you suffer from 'nerves'?  Do you often feel lonely?  Are you often troubled by feelings of guilt?  Participants could answer Yes, No, Do not know or Prefer not to answer.  This field summarises the number of Yes answers across these twelve questions into a single integer score for each participant.  This derived data field is made available by the UK Biobank as provided from Professor Jill Pell from the Institute of Health & Wellbeing, University of Glasgow |

**Supplementary Table 2.** Sensitivity analyses smoking status

|  | **Current smoker** | | | **WOMEN: Current smoker** | | | **MEN: Current smoker** | | |
| --- | --- | --- | --- | --- | --- | --- | --- | --- | --- |
| *Variable* | *Odds Ratios* | *95% CI* | *p-value* | *Odds Ratios* | *95% CI* | *p-value* | *Odds Ratios* | *95% CI* | *p-value* |
| Femininity score | 0.96 | 0.95 – 0.97 | <.001 | 0.95 | 0.94 – 0.97 | <.001 | 0.97 | 0.95 – 0.99 | 0.001 |
| Sex | 1.39 | 1.36 – 1.43 | <.001 |  |  |  |  |  |  |
| Age | 0.97 | 0.97 – 0.97 | <.001 | 0.97 | 0.97 – 0.97 | <.001 | 0.97 | 0.97 – 0.98 | <.001 |
| Observations | 315,223 | | | 166,344 | | | 148,879 | | |

**Supplementary Table 3.** Sensitivity analyses 'vitamin supplement use'

|  | **Vitamin supplement use** | | | **WOMEN: Vitamin supplement use** | | | **MEN: Vitamin supplement use** | | |
| --- | --- | --- | --- | --- | --- | --- | --- | --- | --- |
| *Variable* | *Odds Ratios* | *95% CI* | *p-value* | *Odds Ratios* | *95% CI* | *p-value* | *Odds Ratios* | *95% CI* | *p-value* |
| Femininity score | 0.99 | 0.98 – 0.99 | 0.001 | 0.98 | 0.97 – 0.99 | <.001 | 1.00 | 0.99 – 1.01 | 0.86 |
| Sex | 0.65 | 0.64 – 0.66 | <.001 |  |  |  |  |  |  |
| Age | 1.01 | 1.01 – 1.01 | <.001 | 1.01 | 1.01 – 1.01 | <.001 | 1.01 | 1.01 – 1.01 | <.001 |
| Observations | 315,250 | | | 166,472 | | | 148,778 | | |

**Supplementary Table 4.** Logistic regression for angina diagnosis prior to the occurrence of MI in individuals with incident MI and no history of MI at baseline. In upper and lower age groups, cut-off is median age in individuals with incident MI.

|  | **Women age ≤ 62 years** | | | **Women age > 62 years** | | | **Men age ≤ 62 years** | | | **Men age > 62 years** | | |
| --- | --- | --- | --- | --- | --- | --- | --- | --- | --- | --- | --- | --- |
| *Variable* | *Odds Ratios* | *95% CI* | *p-value* | *Odds Ratios* | *95% CI* | *p-value* | *Odds Ratios* | *95% CI* | *p-value* | *Odds Ratios* | *95% CI* | *p-value* |
| Femininity score | 0.80 | 0.57 - 1.14 | 0.22 | 1.03 | 0.76 - 1.41 | 0.83 | 1.20 | 1.01 - 1.41 | 0.03 | 1.48 | 1.25 - 1.76 | <.001 |
| Age | 1.06 | 0.98 - 1.14 | 0.15 | 1.01 | 0.90 - 1.14 | 0.84 | 1.05 | 1.02 - 1.08 | 0.002 | 1.10 | 1.02 - 1.19 | 0.01 |
| Observations | 404 |  |  | 410 | | | 1,394 | | | 1,084 | | |

**Supplementary Table 5.** Logistic regression for angina diagnosis prior to the occurrence of MI in individuals with incident MI and no history of MI at baseline. In upper and lower deprivation index groups, cut-off is mean Townsend Deprivation Index.

|  | **Women TDI < -1.56** | | | **Women TDI ≥ -1.56** | | | **Men TDI < -1.56** | | | **Men TDI ≥ -1.56** | | |
| --- | --- | --- | --- | --- | --- | --- | --- | --- | --- | --- | --- | --- |
| *Variable* | *Odds Ratios* | *95% CI* | *p-value* | *Odds Ratios* | *95% CI* | *p-value* | *Odds Ratios* | *95% CI* | *p-value* | *Odds Ratios* | *95% CI* | *p-value* |
| Femininity score | 0.80 | 0.57 - 1.12 | 0.20 | 1.09 | 0.79 - 1.50 | 0.61 | 1.43 | 1.20 - 1.69 | <.001 | 1.20 | 1.02 - 1.42 | 0.03 |
| Age | 1.12 | 1.05 - 1.19 | <.001 | 1.08 | 1.03 - 1.13 | <.001 | 1.09 | 1.06 - 1.13 | <.001 | 1.04 | 1.02 - 1.07 | <.001 |
| Observations | 433 |  |  | 381 | | | 1,436 | | | 1,042 | | |
| TDI: Townsend deprivation index | | |  |  |  |  |  |  |  |  |  |  |
